# Publication and related biases in health services research: a systematic review of empirical evidence

**DOI:** 10.1186/s12874-020-01010-1

**Published:** 2020-06-01

**Authors:** Abimbola A. Ayorinde, Iestyn Williams, Russell Mannion, Fujian Song, Magdalena Skrybant, Richard J. Lilford, Yen-Fu Chen

**Affiliations:** 1 Warwick Centre for Applied Health Research & Delivery, Division of Health Sciences, Warwick Medical School, University of Warwick, Coventry, UK; 2 Health Services Management Centre, School of Social Policy, University of Birmingham, Birmingham, UK; 3 Norwich Medical School, University of East Anglia, Norwich, UK; 4 Institute of Applied Health Research, University of Birmingham, Birmingham, UK

**Keywords:** Publication bias, Outcome reporting bias, Dissemination bias, Grey literature, Research publication, Research registration, Health services research, Systematic review, Research methodology, Funnel plots

## Abstract

**Background:**

Publication and related biases (including publication bias, time-lag bias, outcome reporting bias and p-hacking) have been well documented in clinical research, but relatively little is known about their presence and extent in health services research (HSR). This paper aims to systematically review evidence concerning publication and related bias in quantitative HSR.

**Methods:**

Databases including MEDLINE, EMBASE, HMIC, CINAHL, Web of Science, Health Systems Evidence, Cochrane EPOC Review Group and several websites were searched to July 2018. Information was obtained from: (1) Methodological studies that set out to investigate publication and related biases in HSR; (2) Systematic reviews of HSR topics which examined such biases as part of the review process. Relevant information was extracted from included studies by one reviewer and checked by another. Studies were appraised according to commonly accepted scientific principles due to lack of suitable checklists. Data were synthesised narratively.

**Results:**

After screening 6155 citations, four methodological studies investigating publication bias in HSR and 184 systematic reviews of HSR topics (including three comparing published with unpublished evidence) were examined. Evidence suggestive of publication bias was reported in some of the methodological studies, but evidence presented was very weak, limited in both quality and scope. Reliable data on outcome reporting bias and p-hacking were scant. HSR systematic reviews in which published literature was compared with unpublished evidence found significant differences in the estimated intervention effects or association in some but not all cases.

**Conclusions:**

Methodological research on publication and related biases in HSR is sparse. Evidence from available literature suggests that such biases may exist in HSR but their scale and impact are difficult to estimate for various reasons discussed in this paper.

**Systematic review registration:**

PROSPERO 2016 CRD42016052333.

## Background

**Fig. 1:**
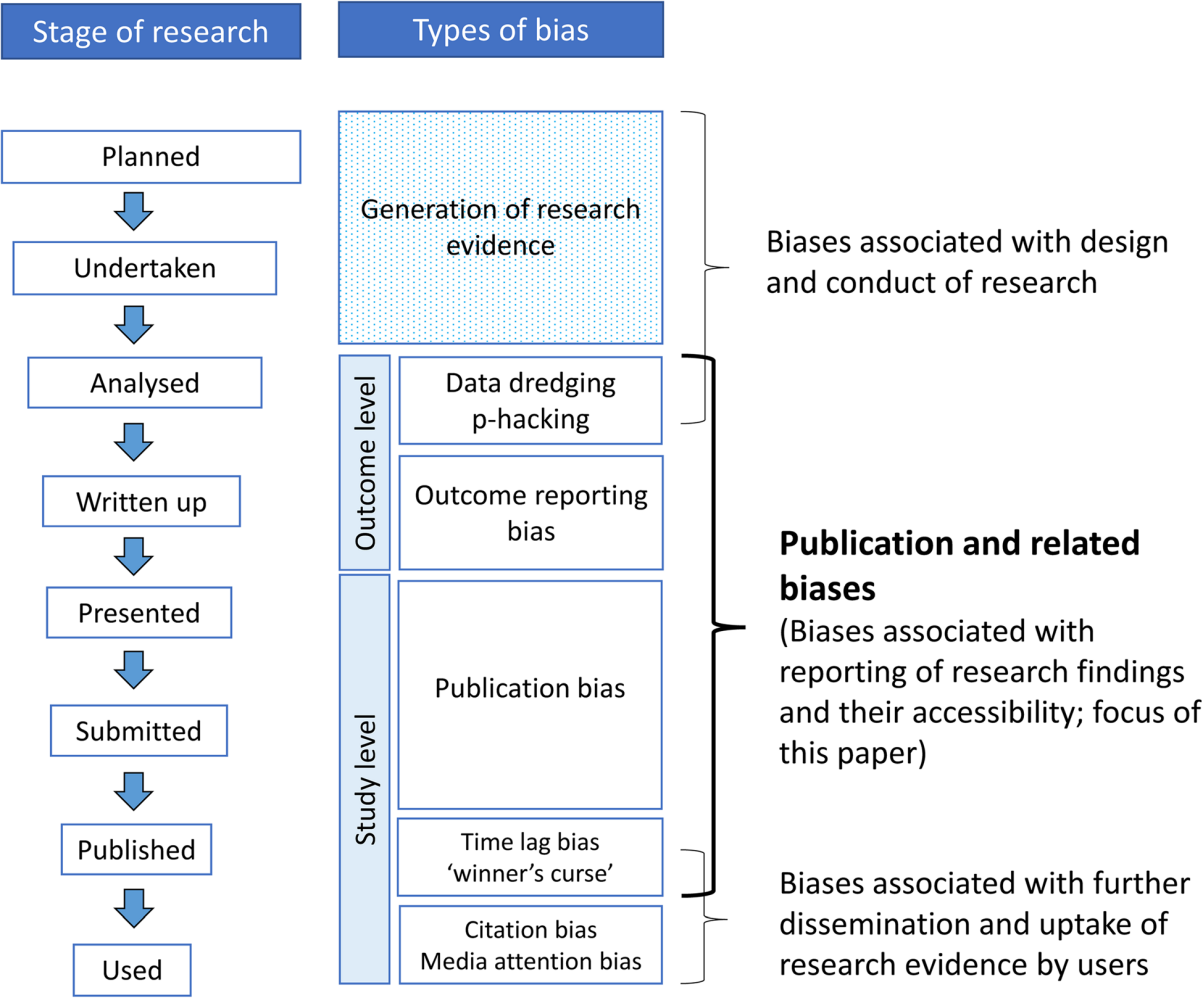
Publication related biases and other biases at various stages of research

Publication bias occurs when the publication, non-publication or late publication of research findings is influenced by the direction or strength of the results, and consequently the findings that are published or published early may differ systematically from those that remain unpublished or for which publication is delayed [1, 2]. Other related biases, however, may occur between the generation of research evidence and its eventual publication. These include: p-hacking, which involves repeated analyses using different methods or subsets of data until statistically significant results are obtained [3]; and outcome reporting bias, whereby among those examined, only favourable outcomes are reported [4]. For brevity, we use the term “publication and related bias” in this paper to encompass these various types of biases (Fig. 1).

Publication bias is a major concern in health care as biased evidence available to decision makers may lead to suboptimal decisions that a) negatively impact on the care and the health of patients and b) lead to an inefficient and inequitable allocation of scarce resources. This problem has been documented extensively in the clinical research literature [2, 4, 5], and several high-profile cases of non-publication of studies showing unfavourable results have led to the introduction of mandatory prospective registration of clinical trials [6]. By comparison, publication bias appears to have received scant attention in health services research (HSR). A recent methodological study of Cochrane reviews of HSR topics found that less than one in 10 of the reviews explicitly assessed publication bias [7].

However, it is unlikely that HSR is immune from publication and related biases, and these problems may be anticipated on theoretical grounds. In contrast with clinical research, where mandatory registration of all studies involving human subjects has long been advocated through the declaration of Helsinki [8] and publication of results of commercial trials are increasingly enforced by regulatory bodies, the registration and regulation of HSR studies are much more variable. In addition, studies in HSR often examine a large number of factors (independent variables, mediating variables, contextual variables and outcome variables) along a long service delivery causal chain [9]. The scope for ‘data dredging’ associated with use of multiple subsets of data and analytical techniques is substantial [10]. Furthermore, there is a grey area between research and non-research, particularly in the evaluation of quality improvement projects [11], which are usually initiated under a service imperative rather than to produce generalizable knowledge. In these settings there are fewer checks against the motivation that may arise post hoc to selectively publish “newsworthy” findings from evaluations showing promising results.

The first step towards improving our understanding of publication and related biases in HSR, which is the main aim of this review, is to systematically examine the existing literature. We anticipated that we might find two broad types of literature: (1) methodological research that set out with the prime purpose of investigating publication and related bias in HSR; (2) systematic reviews of substantive HSR topics but in which the authors had investigated the possibility of publication and related biases as part of the methodology used to explore the validity of their findings.

## Methods

### Scope

We adopted the definition of HSR used by the United Kingdom’s National Institute for Health Research Health Services & Delivery Research (NIHR HS & DR) Programme: “research to produce evidence on the quality, accessibility and organisation of health services”, including evaluation of how healthcare organizations might improve the delivery of services. The definition is deliberately broad in recognition of the many associated disciplines and methodologies, and is compatible with other definitions of HSR such as those offered by the Agency for Healthcare Research and Quality (AHRQ). We were aware that publication bias may arise in qualitative research [12], but as the mechanisms and manifestations are likely to be very different, we focused on publication bias related to quantitative research in this review. The protocol for this systematic review was pre-registered in the PROSPERO International prospective register of systematic reviews (2016:CRD42016052333). We followed the PRISMA statement [13] for undertaking and reporting this review where applicable (see Additional file 1 for the PRISMA checklist).

### Inclusion criteria

Included studies needed to be concerned with HSR related topics based on the NIHR HS & DR Programme’s definition described above. The types of study included were either:

- (1) methodological studies that set out to investigate data dredging/p-hacking, outcome reporting bias or publication bias by one or more of: a) tracking a cohort of studies from inception or from a pre-publication stage such as conference presentation to publication (or not); b) surveying researchers about their experiences related to research publication; c) investigating statistical techniques to prevent, detect or mitigate the above biases;
- (2) systematic reviews of substantive HSR topics that provided empirical evidence concerning publication and related biases. Such evidence could take various forms such as comparing findings in published vs. grey literature; statistical analyses (e.g. funnel plots and Egger’s test); and assessment of selective outcome reporting within individual studies included in the reviews.

### Exclusion criteria

Articles were excluded if they assessed publication and related biases in subject areas other than HSR (e.g. basic sciences; clinical and public health research) or publication bias purely in relation to qualitative research. Biases in the dissemination of evidence following research publication, such as citation bias and media attention bias, were not included since they can be alleviated by systematic search [2]. Studies of bias relating to study design (such as recall bias) were also excluded. No language restriction was applied.

### Search strategy

We used a judicious combination of information sources and searching methods to ensure that our coverage of the relevant HSR literature was as comprehensive as possible. MEDLINE (1946 to 16 March 2017), EMBASE (1947 to 16 March 2017), Health Management Information Consortium (HMIC, 1979 to January 2017), CINAHL (1981 to 17 March 2017), and Web of Science (all years) were searched using indexed terms and text words related to HSR [14], combined with search terms relating to publication bias. In April 2017 we searched HSR-specific databases including Health Systems Evidence (HSE) and the Cochrane Effective Practice and Organisation of Care (EPOC) Review Group using publication bias related terms. The search strategy for MEDLINE is provided in Appendix 1 (see Additional file 2).

For the included studies, we used forward and backward citation searches (using Google Scholar/PubMed and manual check of reference lists) to identify additional studies that had not been captured in the electronic database searches. We searched the webpages of major organizations related to HSR, including the Institute for Healthcare Improvement (USA), The AHRQ (USA), and the Research and Development (RAND) Corporation (USA), Health Foundation (UK), King’s Fund (UK) (last searched on 20th September 2017). We also searched the UK NIHR HSDR Programme website and the US HSRProj (Health Services Research Projects in Progress) database for previously commissioned and ongoing studies (last searched on 20th February 2018). All the searches were updated between 30th July and 2nd August 2018 in order to identify any new relevant methodological studies. Members of the project steering and management committees were consulted to identify any additional studies.

Citations retrieved were imported and de-duplicated in the EndNote software, and were screened for relevance based on titles and abstracts. Full-text publications were retrieved for potentially relevant records and articles were included/excluded based on the selection criteria described above. The screening and study selection were carried out by two reviewers independently, with any disagreement resolved by discussion with the wider research team.

### Data extraction

#### Methodological studies

For the included methodological studies set out to examine publication and related biases, a data extraction form was designed to collect the following information: citation details; methods of selecting study sample; characteristics of study sample; methods of investigating publication and related biases; key findings; limitations; and conclusions. Data extraction was conducted by one reviewer and checked by another reviewer.

#### Systematic reviews of substantive topics of HSDR

For systematic reviews that directly compared published literature with grey literature/unpublished studies, the following data were collected by one reviewer and checked by another: the topic being examined; methods used to identify grey literature and unpublished studies; findings of comparisons between published and grey/unpublished literature; limitations and conclusions. A separate data extraction form was used to collect data from the remaining HSR systematic reviews. Information concerning techniques used to investigate publication bias and outcome reporting bias was extracted along with findings of these investigations. Due to the large number of identified HSR systematic reviews falling into this category, the data extraction was carried out only by a single reviewer.

### Risk of bias assessment

No single risk of bias assessment tool could capture the dimensions of quality for the types of methodological studies included [2]. We therefore critically appraised individual methodological studies and systematic reviews directly comparing published vs unpublished evidence on the basis of adherence to commonly accepted scientific principles, including: representativeness of published/unpublished HSR studies being examined or health services researchers being surveyed; rigour in data collection and analysis; and whether attention was paid to factors that could confound the association between study findings and publication status. Each study was read by at least two reviewers and any methodological issues identified are presented as commentary alongside study findings in the results section. No quality assessment was carried out for the remaining HSR systematic reviews, as we were only interested in their findings in relation to publication and related biases rather than the effects or associations examined in these reviews per se. We anticipated that it would not be feasible to use quantitative methods (such as funnel plots) for evaluating potential publication bias across studies due to heterogeneous methods and measures adopted to assess publication bias in the methodological studies included in this review.

### Data synthesis and presentation

As included studies used diverse approaches and measures to investigate publication and related biases, meta-analyses could not be performed. Findings were therefore presented narratively [15].

## Results

### Literature search and selection

**Fig. 2:**
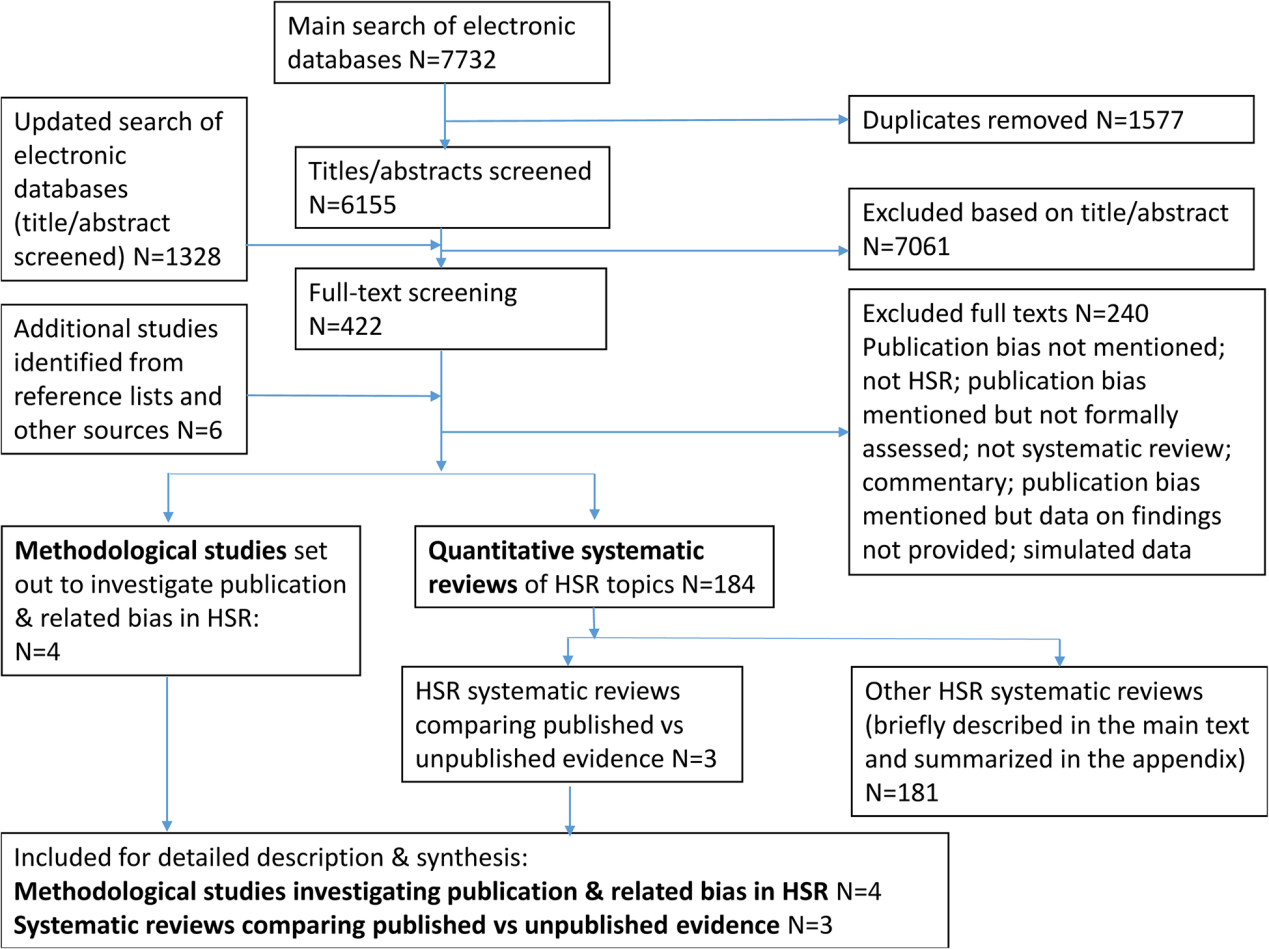
Flow diagram showing study selection process

The initial searches of the electronic databases yielded 6155 references, which were screened on the basis of titles/abstracts. The full-text for 422 of them and six additional articles identified from other sources were then retrieved and assessed (Fig. 2). Two hundred and forty articles did not meet the inclusion criteria primarily because no empirical evidence on publication and related biases was reported or the subject areas lay outside the domain of HSR as described above. An updated search yielded 1328 new records but no relevant methodological studies were identified.

We found four methodological studies that set out with the primary purpose of investigating publication and related biases in HSR [16–19]. We identified 184 systematic reviews of HSR topics where the authors of reviews looked for evidence of publication and related biases. Three of these 184 systematic reviews provided direct evidence on publication bias by comparing findings of published articles with those of grey literature and unpublished studies [20–22]. The remaining 181 review provided only indirect evidence on publication and related biases (Fig. 2).

### Methodological studies setting out to investigate publication and related biases

The characteristics of the four included methodological studies are presented in Table 1. Three studies [16, 17, 19] explored the presence or absence of publication bias in health informatics research. The remaining study [18] focused on p-hacking or reporting bias that may arise when authors of research papers compete by reporting ‘more extreme and spectacular results’ in order to optimize chances of journal publication. A brief summary of each of the studies is provided below.

**Table 1:** Characteristics of included methodological studies investigating publication bias in HSR

Study (HSDR Topic)	Objectives	Methods	Key Findings	Limitations
Ammenwerth, 2007 [16] (Health Informatics)	To determine: - what percentage of IT evaluation studies are not published in international journals or proceedings - what are typical reasons for not publishing the results of an IT evaluation study	Written, e-mail-based survey of academics. Survey sample included members of several mailing lists and first authors of IT evaluation papers that were published between 2001 and 2006 and Medline indexed	Only half of the evaluation studies reported by responders were published. Common reasons for non-publication included ‘not of interest for others’, ‘no time for writing’, ‘limited scientific quality’, ‘political and legal reasons’ and ‘only meant for internal use’	Low response rate (19%, 136/722). Study could be influenced by sampling, response and recall bias
Costa-Font, 2013 [18] (Health Policy)	To examine the winner’s curse phenomenon (studies needing to have more extreme results to be published in high-impact journals) and publication selection bias using quantitative findings on income and price elasticities as reported in health economics research	Funnel plot and multivariate analysis to examine the association between estimated effect sizes (and their statistical significance) and the impact factors of the journals in which they are published	Meta-regression analysis demonstrated that both publication bias (reflected by positive correlation between effect size and standard error) and the winner’s curse (reflected by an independent association between effect size and journal impact factor) influence the estimated income/price elasticity	Alternative explanations for the observed associations cannot be excluded. Literature in the field concerned are often reported in grey literature rather than academic journals
Machan, 2006 [17] (Health Informatics)	To determine: - the percentage of evaluation studies describing positive, mixed or negative results -the possibility of statistical assessment of publication bias in health informatics - the quality of reviews and meta-analysis in health informatics with regard to publication bias	Descriptive analysis of random sample of 86 evaluation studies and planned to construct funnel plot Examined characteristics and quality of reviews and meta-analyses (*n* = 54) in medical informatics	For the primary studies, 69.8% had positive results, 14% negative and 16.3% unclassified For the reviews 36.6% had positive conclusion, 61.5% were inconclusive, and only one review came to a negative conclusion	Small number of comparable studies prevented the quantitative analysis of potentiation publication bias Proportion of studies/reviews with a positive conclusion may not be good indicators for the existence of publication bias
Vawdrey, 2013 [19] (Health Informatics)	To measure the rate of non-publication and assess possible publication bias in clinical trials of electronic health records	Follow-up of health informatics trials registered in ClinicalTrials.gov (2000–2008)	Trials with positive results were more likely to be published compared with trials with null results (92% of trials with positive results [35/38] vs 75% of trials with neutral or negative results [12/16], but the difference was not statistically significant (*p* = 0.177^a^)	Sample size relatively small; no information could be obtained for 8 unpublished trials; completeness of trial registration and representativeness of registered uncertain

^a^ Fisher’s exact test. Authors of the original article presented their data according to whether trials were published or unpublished; findings presented here are based on the same data but are organised according to whether the findings of the trials were positive or neutral/negative. We believe this is a more suitable presentation of the data, as the hypothesis is the probability of publication being conditional upon positivity of the trial, not the other way round

Only one study was an inception cohort study, which tracked individual research projects from their start. Such a study provides direct evidence of publication bias [19]. This study assessed publication bias in clinical trials of electronic health records registered with ClinicalTrials.gov during 2000–8 and reported that results from 76% (47/62) of completed trials were subsequently published. Of the published studies, 74% (35/47) reported predominantly positive results, 21% (10/47) reported neutral results (no effect) and 4% (2/47) reported negative/harmful results. Data were available from investigators for seven of the 15 unpublished trials: four reported neutral results and three reported positive results. Based on these data, the authors concluded that trials with positive results are more likely to be published than those with null results, although we noticed that this finding was not statistically significant (see Table 1). The authors cautioned that few trials were registered in the early years of ClinicalTrials.gov and those registered may be more likely to publish their findings and thus systematically different from those not registered. They further noted that the registered data were often unreliable during that period.

The second study reported a pilot survey of academics in order to assess rates of non-publication in IT evaluation studies and reasons for any non-publication [16]. The survey asked what information systems the respondents had evaluated in the past 3 years, whether the results of the evaluation(s) were published, and if not published, the reasons behind the non-publication. The findings show that approximately 50% of the identified evaluation studies were published in peer reviewed journals, proceedings or books. Of the remaining studies, some were published in internal reports and/or local publications (such as masters’ theses and local conferences) and approximately one third were unpublished at the time of the survey. The reasons cited for non-publication included: “results not of interest for others”; “publication in preparation”; “no time for publication”; “limited scientific quality of study”; “political or legal reasons”, and “study only conducted for internal use”. The main limitation of this study is a low response rate with only 118 of 722 (18.8%) targeted participants providing valid responses.

The third methodological study used three different approaches to assess publication bias in health informatics [17]. However, for one of the approaches (statistical analyses of publication bias/small study effects) the authors were unable to find enough studies which reported findings using the same outcome measures; while the remaining two approaches adopted in this study (i.e. examining percentage of HSR evaluation studies reporting positive results and percentage of HSR reviews reaching positive conclusion) provided little information on publication bias since there is no estimate of what the “unbiased” proportion of positive findings should be for HSR evaluation studies and reviews (Table 1).

The fourth methodological study included in this review examined quantitative estimates of income elasticity of health care and price elasticity of prescription drugs reported in the published literature [18]. Using funnel plots and meta-regressions the authors identified a positive correlation between effect sizes and the standard errors of income/price elasticity estimates, which suggested potential publication bias [18]. In addition, they found an independent association between effect size and journal impact factor, indicating that given similar standard errors (which reflect sample sizes), studies reporting larger effect sizes (i.e. more striking findings) were more likely to be published in ‘high-impact’ journals. As other confounding factors could not be ruled out for these observed associations and no unpublished studies were examined, the evidence is suggestive rather than conclusive.

### Systematic reviews of HSR topics providing evidence on publication and related bias

We identified 184 systematic reviews of HSR topics in which empirical evidence on publication and related bias was reported. Three of these reviews provided direct evidence on publication bias by comparing evidence from studies published in academic journals with those from grey literature or unpublished studies [20–22]. These reviews are described in detail in the next sub-section. The remaining 181 reviews only provided indirect evidence and are summarised briefly in the subsequent sub-section and in Appendix 2 (see Additional file 2).

#### HSR systematic reviews comparing published and grey/unpublished evidence

Three HSR systematic reviews made such comparisons [20–22]. The topics of these reviews and their findings are summarised in Table 2. The first review evaluated the effectiveness of mass mailings for increasing the utilization of influenza vaccine [22], focusing on evidence from controlled trials. The authors found one published study reporting statistically significant intervention effects, but additionally identified five unpublished studies through a Medicare quality improvement project database. All the unpublished studies reported clinically trivial intervention effects (no effect or an increase of less than two percentage point in uptake). This case illustrated the practical implications of publication bias: the authors highlighted that further mass mailing interventions were being considered by service planners on the basis of results from the first published study when they presented the review findings.

**Table 2:** HSR systematic reviews that have compared literature published literature with grey/unpublished literature

Study (HSDR Topic)	Topic	Methods of identifying grey literature/unpublished studies	Key Findings of comparison between published literature and grey literature/unpublished studies	Limitations
Maglione, 2002 [22] (Immunization Program)	Effectiveness of mass mailings to increase utilization of influenza vaccine among Medicare beneficiaries	Search of the Medicare Peer Review Organization Health Care Quality Improvement Project database	Six controlled trials were identified. Only one (earliest) trial reporting modest but statistically significant improvement in vaccination rate (2–8% depending on the format of the letter and location of the study) was published. Five subsequent trials which found smaller, clinically trivial improvement in vaccination rate of no more than 2% remained unpublished	The review only included a small number of trials identified from a single study registry and targeting a specific US population
Batt,2004 [20] (Immunization program)	Costs, effects and cost-effectiveness of strategies to increase coverage of routine immunizations in low- and middle income countries	Hand searches in institutional documentation centres including WHO and USAID; interviews with 28 international experts; search of grey literature databases; searches of the internet, conference proceedings and webpages of pertinent organizations	Quality of data on effect and cost-effectiveness was similar between published and grey literature, but the quality of costing data was poorer in grey literature. Inclusion of grey literature doubled the quantity of available evidence. Interventions examined in the grey literature were more up to date, associated with more complex interventions aimed at health systems and better represented west Africa and the Middle East. Conclusions drawn from the two sets of literature therefore differed	Reviewed grey literature was mainly derived from international organizations with little coverage of national governments. Searches were limited to English keywords
Fang, 2007 [21] (Organizational studies)	Relationships between organizational culture, organizational climate, and nurse’s job satisfaction and turnover	Extensive search of 35 databases, “footnote chasing”, and searching by author	Of the nine associations for which findings were compared between published articles and unpublished doctoral dissertations, significant differences were found for three of them: association between passive/defensive culture and job satisfaction; global climate and job satisfaction; and reward orientation climate and job satisfaction. All the differences were related to magnitude rather than direction of the estimated association	Grey literature was limited to doctoral dissertations. The number of studies was very small for some of the comparisons; in some cases only one published or unpublished study was available

The second review compared the grey literature [20] with published literature [23] on the effectiveness and cost-effectiveness of strategies to improve immunization coverage in developing countries, and found that the quality and nature of evidence differed between these two sources of evidence, and that the recommendations about the most cost-effective interventions would differ between the two reviews (Table 2).

The third review assessed nine associations between various measures of organisational culture, organisational climate and nurse’s job satisfaction [21]. The author included both published literature and doctoral dissertations in the review, and statistically significant differences in the pooled estimates between these two types of literature were found in three of the nine associations (Table 2).

#### Findings from other systematic reviews of HSR topics

Of the 181 remaining systematic reviews, 100 examined potential publication bias *across* studies included in the reviews using funnel plots and related techniques, and 108 attempted to assess outcome reporting bias *within* individual included studies, generally as part of the risk of bias assessment. The methods used in these reviews and key findings in relation to publication bias and outcome reporting bias are summarised in Appendix 2 (see Additional file 2). Fifty-one of the 100 reviews which attempted to assess publication bias showed some evidence of its existence (through the assumption that observed small study effects were caused by publication bias).

For the assessment of outcome reporting bias, reviewers frequently reported difficulties in judging outcome reporting bias due to the absence of a published protocol for the included studies. For instance, a Cochrane review of the effectiveness of interventions to enhance medication adherence included 182 RCTs and judged eight and 32 RCTs to be of high and low risk for outcome reporting bias respectively, but the remaining 142 RCTs were judged to be of unclear risk, primarily due to unavailability of protocols [24]. In the absence of a protocol, some reviewers assessed outcome reporting bias by comparing outcomes specified in the methods to those presented in the results section, or made subjective judgements on the extent to which all important outcomes were reported. However, the validity of such approaches remains unclear. All but one of the reviews that assessed outcome reporting bias used either the Cochrane risk of bias tool (the checklist developed by the Cochrane Collaboration for assessing internal validity of individual RCTs) or bespoke tools derived from this. The remaining review - of the effectiveness of interventions for hypertension care in the community - undertook a sensitivity analysis to explore the influence of studies that otherwise met the inclusion criteria except for not providing sufficient data on relevant outcomes [25]. This was achieved by imputing zero effects (with average standard deviations) for the studies with missing outcomes (40 to 49% of potentially eligible studies), including them in the meta-analysis and recalculating the pooled effect. They found that the pooled effect was considerably reduced although still statistically significant [25]. These reviews illustrate the challenges of assessing outcome reporting bias in HSR and in identifying its potential consequences.

Delay in publication arising from the direction or strength of the study findings, referred to as time lag bias, was assessed in one of the reviews which evaluated the effectiveness of interventions for increasing the uptake of mammography in low and middle income countries [26]. The authors classified the time lag from end of intervention to the publication date into ≤4 years and > 4 years and reported that studies published within 4 years showed stronger association between intervention and mammography uptake (risk differences: 0.10, 95% CI 0.08, 0.12) when compared to studies published more than 4 years after completion (0.08, 95% CI 0.04, 0.11). However, the difference between the two subgroups was very small and not statistically significant (F ratio = 2.94, *p* = 0.10), and it was not clear whether this analysis and the cut-off time lag for defining the subgroups were specified a priori.

## Discussion

This systematic review examined current empirical evidence on publication and related biases in HSR. Very few methodological studies that directly investigated these issues were found. Nonetheless, a small number of available studies focusing on publication bias suggested its existence: findings of studies were not always reported/published; those published were often with positive results, and were sometimes of different nature, which could impact upon their applicability and relevance for different users of the evidence. There was also evidence suggesting that studies reporting larger effect sizes were more likely to be published in high impact journals. However, there are methodological weaknesses behind these pieces of evidence, which does not allow a firm conclusion to be drawn.

Reasons for non-publication of HSR findings described in the only survey we found appear to be similar to those of clinical research [27]. Lack of time and interest from the part of the researcher appears to be a major factor, which could exacerbate when the study findings are uninteresting. Also of note are comments such as “not of interest for others” and “only meant for internal use”. These not only illustrate context-sensitive nature of evidence for HSR, but also highlight issues arising from the hazy boundary between research and non-research for many evaluations undertaken in healthcare organizations, such as quality improvement projects and service audits. As promising findings are likely to motivate publication of these quality improvement projects, caution is required in interpreting and particularly in generalizing their findings. Another reason given for non-publication in HSR is “political and legal reasons”. Publication bias and restriction of access to data arising from conflict of interest is well documented in clinical research [2] and one might expect similar issues in HSR. We did not identify methodological research specifically related to the impact of conflict of interest on publication of findings in HSR, although anecdotal evidence of financial arrangement influencing editorial process exists [28], and there are debates concerning public’s accessibility of information related to health services and policy [29].

It is currently difficult to gauge the true scale and impact of publication and related biases given the sparse high quality evidence. Among the four methodological studies identified in this review, only one was an inception cohort study that provided direct evidence. This paucity of evidence is in stark contrast with a methodological review assessing publication bias and outcome reporting bias in clinical research, in which 20 inception cohort studies of RCTs were found [4]. The difference between these two fields is likely to be in part attributable to the less frequent use of RCTs in HSR and lack of requirement for study registration. The lesser reliance on RCTs and lack of study registration present a major methodological challenge in studying publication bias in HSR as there is no reliable way to identify studies that have been conducted but not subsequently published.

The lack of prospective study registration poses further challenges in assessing outcome reporting bias, which could be a greater concern for HSR than clinical research given the more exploratory approaches to examining a larger number of variables and associations in HSR. Empirical evidence on selective outcome reporting has primarily been obtained from RCTs as study protocols are made available in the trial registration process [4]. Calls for prospective registration of study protocols of observational studies have been made [30] and repositories of quality improvement projects are emerging [31]. HSR and quality improvement communities will need to consider and evaluate the feasibility and values of adopting these practices.

Statistical techniques such as funnel plots and regression methods are commonly used in HSR systematic reviews to identify potential publication bias, as in clinical research. Assumptions (e.g. any observed small study effects are caused by publication bias) and conditions (e.g. at least 10 studies measuring the same effect) related to the appropriate use of these techniques hold true for HSR, but heterogeneity commonly found among HSR studies resulting from the inherent complexity and variability of service delivery interventions and their interaction with contextual factors [32, 33] may further influence the validity of funnel plots and related methods [34], and findings from these methods should be treated with caution [35].

In addition to the conventional methods discussed above, new methods such as p-curves for detecting p-hacking have emerged in recent years [36, 37]. P-curves have been tested in various scientific disciplines [3, 38, 39], although no studies that we examined in the field of HSR have used this technique. The validity and usefulness of p-curves are subject to debate and accumulation of further empirical evidence [40–43].

Given the limitations of statistical methods, search of grey literature and contacting stakeholders to unearth unpublished studies remain an important means of mitigating publication bias, although this is often resource intensive and does not completely eliminate the risk. The finding from Batt et al. (2004) described above highlighted that published and grey literature could differ in their geographical coverage and nature of evidence [20]. This has important implications given the context-sensitive nature of HSR.

The limited evidence that we found does not allow us to estimate precisely the scale and impact of publication and related biases in HSR. It may be argued that publication bias may not be as prevalent in HSR as in clinical research because of the complexity of health systems which makes it often necessary to investigate the associations between a large number of variables along the service delivery causal pathway. As a result, HSR studies may be less likely to have completely null results or to depend for their contribution on single outcomes. Conversely, this heterogeneity and complexity may increase the scope for p-hacking and outcome reporting bias in HSR, which are even more difficult to prevent and detect.

A major challenge for this review was to delineate a boundary between HSR and other health/medical research. We used a broad range of search terms and identified a large number of studies, many of which were subsequently excluded after screening. We have used the definition of HSR provided by the UK NIHR and therefore our review may not have covered some areas of HSR if defined more broadly. We combined publication bias related terms with HSR related terms in our searches. As a result, we might not have captured some HSR related studies which have investigated publication and related bias but which did not mention them in their titles, abstracts or indexed terms. This is most likely to occur for systematic reviews of substantive HSR topics, in which funnel plot and related methods might have been deployed as a routine procedure to examine potential publication bias. Nevertheless, it is well known that statistical techniques such as funnel plot and related tests have low statistical power, and publication bias is just one of the many potential reasons behind ‘small study effects’ which these methods actually detect [34]. Findings from these systematic reviews are therefore of limited value in terms of confirming or refuting the existence of publication bias. Despite the limitation related to the search strategy, we identified and briefly examined more than 180 systematic reviews as shown in Appendix 2 in the supplementary file, but except for the small number of systematic reviews highlighted in the Results section, very little conclusion in relation to publication bias could be drawn from these reviews.

A further limitation of this study is that we have focused on publication and related biases related to quantitative studies and have not covered qualitative research, which plays an important role in HSR. It is also worth noting that three of the four included studies relate to the specific sub-field of health informatics which places limits on the extent to which our conclusions can be generalised to other subfields of HSR. Lastly, although we attempted to search several databases as well as grey literature, the possibility that evidence included in this review is subject to publication and related bias cannot be ruled out.

## Conclusion

There is a paucity of empirical evidence and methodological literature addressing the issue of publication and related biases in HSR. While the available evidence suggests the presence of publication bias in this field, its magnitude and impact is yet to be fully explored and understood. Further research evaluating the existence of publication and related biases in HSR, what factors contribute towards their occurrence, their impact and the range of potential strategies to mitigate them, is therefore warranted.

## Supplementary information

**Additional file 1.** PRISMA checklist.

**Additional file 2.** Appendices.

## Data Availability

All data generated and/or analysed during this review are included within this article and its additional files. This systematic review was part of a large project investigating publication and related bias in HSR. The full technical report for the project will be published in the UK National Institute for Health Research (NIHR) Journals Library: https://www.journalslibrary.nihr.ac.uk/programmes/hsdr/157106/#/

## Abbreviations

AHRQ: Agency for Healthcare Research and Quality
EPOC: Effective Practice and Organisation of Care
HSE: Health Systems Evidence
HSR: Health Services Research
NIHR HS & DR Programme: National Institute for Health Research Health Services & Delivery Research Programme
RCTs: Randomised controlled trials
