# Supplementary material for: Publication and related biases in health services research: a systematic review of empirical evidence
Source: BMC Med Res Methodol. 2020 Jun 1;20:137. doi: 10.1186/s12874-020-01010-1 (PMC7268600; doi:10.1186/s12874-020-01010-1)
Supplement: Supplementary file 2 — Additional file 2. Appendices. [file 12874_2020_1010_MOESM2_ESM.pdf]

Appendices

Publication and related biases in health services research: a systematic review of empirical evidence

Authors: Abimbola A Ayorinde, Iestyn Williams, Russell Mannion, Fujian Song, Magdalena Skrybant, Richard J Lilford, Yen-Fu Chen

Appendix 1 Search strategy for MEDLINE

1 *Health Services Research/ or health service$ research.mp. (37371)

2 service delivery.mp. (10383)

3 health system$.mp. (37050)

4 healthcare system$.mp. (15933)

5 Health Policy.mp. or *Health Policy/ (69602)

6 ((health care or healthcare or hospital$ or service$) adj1 (administration or organi$ation or management or

structure or govern* or financ* or account* or accredit* or policy or efficiency)).mp. (75495)

7 exp Primary Health Care/ and (administration or organi$ation or management or structure or govern* or financ* or

account* or accredit* or policy or efficiency).mp. (42274)

8 exp Governing Board/ (7162)

9 Patient Safety.mp. or exp Patient Safety/ (29465)

10 exp Medical Errors/ (99972)

11 ((adverse event$ or infection$ or complication$) adj1 prevent*).mp. (16088)

12 Quality Improvement.mp. or exp Quality Improvement/ (34488)

13 exp Total Quality Management/ or *Risk Management/ or change management.mp. (21623)

14 exp Organizational Culture/ (15289)

15 leadership.mp. or exp Leadership/ (51370)

16 exp Medical Records Systems, Computerized/ (32367)

17 exp Operations Research/ or operational research.mp. (28506)

18 exp "Appointments and Schedules"/ or waiting time.mp. (21419)

19 exp Triage/ or triage.mp. (17463)

20 ((integrat* or pathway or continuity or access* or model*or transition) adj2 (care or service$)).mp. (98373)

21 (exp Health Personnel/ or (staff or professional$ or healthcare worker$ or health care worker$ or health worker$

or workforce$ or nurse$ or doctor$ or physician$ or trainee$ or consultant$ or general practitioner$ or surgeon$ or dentist$ or therapist$ or pharmacist$ or radiologist$ or pathologist$ or technician$ or assistant$).mp.) and (skill$ or training or education or competence or morale or burnout or absenteeism or retention or deploy* or workforce).mp.

(431677)

22 exp Patient Education as Topic/ (79451)

23 exp Patient Satisfaction/ (75527)

24 *Disease Management/ (15255)

25 (variation$ adj2 (care or service$ or outcome$ or mortality or process*)).mp. (3877)

26 ((organization* or organisation*) adj1 performance).mp. (911)

27 implementation science$.mp. (867)

28 *publication$/ (3633)

29 bias$.mp. or "Bias (Epidemiology)"/ (174661)

30 28 and 29 (63)

31 publication bias.mp. or exp Publication Bias/ (10831)

32 (bias$ adj3 (publication$ or disseminat$ or language$ or reporting or grey or gray or citation$ or time delay or time lag or national or country or location or conference or abstract or duplicat$ or multiple publication$)).tw. (10242)

33 ((reference$ or database$ or index$) adj2 bias$).tw. (410)

34 (file adj1 drawer$).tw. (58)

35 (time adj2 (completion or publication)).tw. (2889)

36 unpublished research.tw. (165)

37 (fail$ adj2 publish$).tw. (235)

38 (p-curve$ or p-hack*).tw. (150)

39 (data adj2 dredg*).tw. (45)

40 phishing.tw. (27)

41 non-publication.tw. (73)

42 selective report$.tw. (516)

43 selective non report$.tw. (3)

44 selective non-report$.tw. (3)

45 outcome report$ bias.tw. (110)

46 1 or 2 or 3 or 4 or 5 or 6 or 7 or 8 or 9 or 10 or 11 or 12 or 13 or 14 or 15 or 16 or 17 or 18 or 19 or 20 or 21 or 22 or 23 or 24 or 25 or 26 or 27 (1114231)

47 30 or 31 or 32 or 33 or 34 or 35 or 36 or 37 or 38 or 39 or 40 or 41 or 42 or 43 or 44 or 45 (17513)

48 46 and 47 (1193)

49 remove duplicates from 48 (1150)

Appendix 2: Methods used to investigate publication bias and outcome reporting bias in included systematic reviews

Study ID (Author, Year)	Type of bias assessed*	Funnel plot	Begg's	Egger's	Trim and Fill	Fail-Safe-N	Reported evidence of publication bias	Cochrane (and related) risk of bias tool	Other approach
Abdalla G., 2015	ORB							✓	
Akl A., 2013	ORB							✓	
Algie C.M., 2015	ORB							✓	
Al-Muhandis N., 2011	PB	✓							
Aoyagi Y., 2015	PB	✓		✓			✓		
Arditi C., 2012	PB and ORB	✓					✓	✓	
Aubin M., 2012	ORB							✓	
Badamgarav E., 2003	PB	✓					✓		
Baker R., 2015	ORB							✓	
Ballini L., 2015	ORB							✓	
Balogh R., 2016	ORB							✓	
Barnard S., 2015	ORB							✓	

Study ID (Author, Year)	Type of bias assessed*	Funnel plot	Begg's	Egger's	Trim and Fill	Fail-Safe-N	Reported evidence of publication bias	Cochrane (and related) risk of bias tool	Other approach
Baskerville N.B., 2012	PB	✓	✓	✓			✓		
Berdot S., 2013	PB	✓		✓					
Bos JM., 2016	ORB							✓	
Bosch-Capblanch X., 2011	ORB							✓	
Bradford D.W., 2013	PB								Searched ClinicalTrials.gov completed unpublished studies and ongoing studies
Brasil PE., 2008	PB	✓	✓	✓			✓		
Brocklehurst P., 2013	ORB							✓	
Brown L., 2015	PB and ORB	✓						✓	
Butler M., 2011	ORB							✓	
Campanella P., 2016	PB	✓		✓					
Campbell F., 2016	ORB							✓	
Cappuccio FP., 2004	PB	✓		✓	✓		✓		
Chaillet N., 2007	PB	✓	✓	✓					

Study ID (Author, Year)	Type of bias assessed*	Funnel plot	Begg's	Egger's	Trim and Fill	Fail-Safe-N	Reported evidence of publication bias	Cochrane (and related) risk of bias tool	Other approach
Chang C.W., 2015	PB	✓		✓			✓		
Chen C.C, 2016	PB	✓							
Chen Y, 2016	PB	✓	✓	✓			✓		
Chen Z., 2008	PB	✓	✓	✓	✓		✓		
Chodosh J., 2005	PB	✓		✓			✓		
Chodosh, 2005	PB	✓	✓	✓			✓		
Christensen, 2016	PB and ORB	✓						✓	
Connock M., 2007	PB	✓	✓	✓					
Cook O., 2015	ORB							✓	
Corbett M, 2015	ORB							✓	
Costa-Font J., 2011	PB	✓		✓			✓		
Cui M., 2016	ORB							✓	
Cuijpers P., 2011	PB	✓			✓				
De Luca G., 2008	PB	✓		✓					

Study ID (Author, Year)	Type of bias assessed*	Funnel plot	Begg's	Egger's	Trim and Fill	Fail-Safe-N	Reported evidence of publication bias	Cochrane (and related) risk of bias tool	Other approach
de Lusignan S., 2014	ORB							✓	
de Roten Y., 2013	PB	✓				✓	✓		
Demonceau J., 2013	PB and ORB	✓		✓			✓	✓	
Donald F., 2014	ORB							✓	
Dorresteijn JAN, 2010	ORB							✓	
Dudley L., 2011	ORB							✓	
Dyer TA., 2014	ORB							✓	
Easthall C., 2012	PB	✓							
Fiander M., 2015	ORB							✓	
Fisher E., 2015	ORB							✓	
Fletcher KE., 2011	PB			✓					
Flodgren G., 2010	ORB							✓	
Flodgren G., 2011	ORB							✓	
Flodgren G., 2011	ORB							✓	

Study ID (Author, Year)	Type of bias assessed*	Funnel plot	Begg's	Egger's	Trim and Fill	Fail-Safe-N	Reported evidence of publication bias	Cochrane (and related) risk of bias tool	Other approach
Flodgren G., 2013	ORB							✓	
Forsetlund L., 2009	PB	✓					✓		
Foy R., 2010	PB	✓	✓	✓					
Free C., 2013	PB and ORB	✓						✓	
French SD., 2010	ORB							✓	
Gallagher RW., 2011	PB and ORB	✓			✓	✓		✓	
Gardner MP., 2013	PB and time lag bias	✓		✓			✓		Time lag bias assessed by comparing the time lag between the end of the intervention period and the date of publication (classified into <=4 years and >4 years)
Gemmill M.C., 2007	PB	✓					✓		
Gensichen J., 2006	PB	✓		✓			✓		
Giguère A., 2012	ORB							✓	
Gillaizeau F., 2013	ORB							✓	
Gillies D., 2015	ORB							✓	
Göhler A., 2006	PB	✓	✓	✓	✓		✓		

Study ID (Author, Year)	Type of bias assessed*	Funnel plot	Begg's	Egger's	Trim and Fill	Fail-Safe-N	Reported evidence of publication bias	Cochrane (and related) risk of bias tool	Other approach
Goldzweig CL., 2015	PB		✓	✓					
Gooiker GA, 2010	PB	✓		✓					
Goossens-Laan CA., 2011	PB	✓		✓					
Green CJ., 2010	ORB							✓	
Grobler L., 2015	ORB							✓	
Grochtdreis T., 2015	ORB							✓	
Gurusamy KS., 2014	ORB							✓	
Han H., 2009	PB	✓				✓	✓		
Hata T., 2016	PB	✓		✓					
Hayhurst KP., 2015	PB	✓					✓		
Higginson IJ., 2003	PB	✓					✓		
Hodgkinson B., 2011	ORB							✓	
Horsley T., 2011	ORB							✓	

Study ID (Author, Year)	Type of bias assessed*	Funnel plot	Begg's	Egger's	Trim and Fill	Fail-Safe-N	Reported evidence of publication bias	Cochrane (and related) risk of bias tool	Other approach
Ivers N., 2012	ORB							✓	
Jacobson VJC., 2005	PB	✓							
Jamal MH., 2012	PB	✓		✓			✓		
Jia L., 2014	ORB							✓	
Kahn SR., 2013	PB and ORB	✓					✓	✓	
Karmali KN., 2014	ORB							✓	
Keebler JR., 2016	PB	✓		✓			✓		
Kelley JM., 2014	PB and ORB	✓	✓		✓	✓		✓	
Kerlin MP., 2017	PB and ORB	✓		✓				✓	Macaskill test
Ketelaar NABM., 2011	ORB							✓	
Khangura JK., 2012	ORB							✓	
Khunpradit S., 2011	ORB							✓	
Kilpatrick K., 2015	ORB							✓	
Kim YL., 2005	PB	✓				✓			

Study ID (Author, Year)	Type of bias assessed*	Funnel plot	Begg's	Egger's	Trim and Fill	Fail-Safe-N	Reported evidence of publication bias	Cochrane (and related) risk of bias tool	Other approach
Kroon FP., 2014	PB and ORB	✓						✓	
Lambrinou E., 2012	PB	✓		✓			✓		
Lau D., 2012	PB	✓							Harbord’s test
Laver KE., 2013	ORB							✓	
Lin H., 2014	PB and ORB	✓	✓					✓	
Lu Z., 2012	PB and ORB	✓		✓			✓		Peter's Method<br>To assess the risk of possible outcome reporting bias, reviewers included studies that met the inclusion criteria but did not provide sufficient data on relevant outcomes
Luiza VL., 2015	ORB							✓	
Lutge EE., 2015	ORB							✓	
Lv L., 2012	ORB							✓	
Ma N., 2017	PB	✓							
Maaskant JM., 2015	ORB							✓	
McBain H., 2016	ORB							✓	

Study ID (Author, Year)	Type of bias assessed*	Funnel plot	Begg's	Egger's	Trim and Fill	Fail-Safe-N	Reported evidence of publication bias	Cochrane (and related) risk of bias tool	Other approach
McGowan JL., 2009	ORB							✓	
Ming WK., 2016	ORB							✓	
Mitchell GK., 2015	ORB							✓	
Moja L., 2014	PB and ORB	✓	✓	✓				✓	
Murphy SM., 2015	ORB							✓	
Murthy L., 2012	ORB							✓	
Neumeyer-Gromen A., 2004	PB	✓	✓	✓					
Nglazi MD., 2013	ORB							✓	
Nieuwlaat R., 2014	ORB							✓	
Oliver D., 2000	PB		✓						
Opiyo N., 2015	ORB							✓	
Opiyo N., 2016	ORB							✓	
O'Sullivan JW., 2016	ORB							✓	

Study ID (Author, Year)	Type of bias assessed*	Funnel plot	Begg's	Egger's	Trim and Fill	Fail-Safe-N	Reported evidence of publication bias	Cochrane (and related) risk of bias tool	Other approach
Panagioti M., 2014	PB	✓		✓			✓		
Pande S., 2013	ORB							✓	
Parmelli E., 2012	ORB							✓	
Peñaloza B., 2011	ORB							✓	
Perrier L., 2011	ORB							✓	
Peterson AM., 2003	PB	✓					✓		
Peytremann-Bridevaux I., 2015	PB and ORB	✓						✓	
Rachoin JS., 2012	PB			✓	✓				
Rashidian A., 2015	ORB							✓	
Reeves S., 2013	ORB							✓	
Robotham D., 2016	PB	✓		✓	✓				
Rockers P., 2013	ORB							✓	
Ross MKM, 2012	PB	✓			✓	✓			

Study ID (Author, Year)	Type of bias assessed*	Funnel plot	Begg's	Egger's	Trim and Fill	Fail-Safe-N	Reported evidence of publication bias	Cochrane (and related) risk of bias tool	Other approach
Rotter T., 2010	PB and ORB	✓						✓	
Rubio-Valera, M	PB	✓		✓					
Ruotsalainen JH., 2015	PB and ORB	✓					✓	✓	
Ruppar TM., 2016	PB	✓							Conducted moderator analyses to evaluate whether effect estimates were different based on factors such as year of publication, publication method (eg, journal article, dissertation) among other factors
Saffari M., 2014	PB and ORB	✓	✓	✓			✓	✓	
Salyers MP., 2016	PB	✓		✓					
Santo K., 2016	PB and ORB	✓		✓	✓		✓	✓	
Scott A., 2011	ORB							✓	
Shamliyan TA., 2008	PB			✓	✓				Peter's Method
Shen Y., 2007	PB	✓							Assessed publication bias by examining the relationship between the square root of degrees of freedom and the absolute value of t-statistics. They also ran a difference-in-difference regression using the absolute t-statistics as the dependent variables and square root of degrees of freedom, ownership-focus indicator, and their interaction term as the independent variables

Study ID (Author, Year)	Type of bias assessed*	Funnel plot	Begg's	Egger's	Trim and Fill	Fail-Safe-N	Reported evidence of publication bias	Cochrane (and related) risk of bias tool	Other approach
Shepperd S., 2016	PB and ORB	✓						✓	
Shepperd S., 2016	ORB							✓	
Shojania KG., 2006	PB	✓					✓		Univariate analysis of sample size and effect size
Singh S., 2014	PB	✓		✓	✓		✓		
Smith SM., 2016	ORB							✓	
Stacey D., 2014	PB and ORB	✓						✓	
Stephens M., 2015	ORB							✓	
Stroke Unit Trialists' Collaboration, 1997	PB	✓							Assessed publication bias by calculating how many randomised patients (with a similar baseline event rate as in the review) would have to be recruited from neutral trials (odds ratio=1.0) to render the overall result non-significant to estimate the degree to which conclusions of the review would be overturned by missing neutral trials
Suphanchaimat R., 2016	PB	✓		✓			✓		
Teding van Berkhout E., 2016	PB	✓			✓	✓	✓		
Thakkar J., 2016	PB	✓		✓	✓		✓		

Study ID (Author, Year)	Type of bias assessed*	Funnel plot	Begg's	Egger's	Trim and Fill	Fail-Safe-N	Reported evidence of publication bias	Cochrane (and related) risk of bias tool	Other approach
Thompson Coon J., 2012	ORB							✓	
Thompson RL., 2003	PB	✓					✓		
Toma T., 2014	PB and ORB	✓		✓			✓	✓	
Tongsai S., 2012	PB	✓	✓	✓	✓				
Tricco AC., 2012	ORB							✓	
Tsai AC., 2005	PB	✓		✓			✓		
Tura G., 2013	PB	✓	✓	✓	✓		✓		
Tyson MD., 2016	PB	✓	✓				✓		
Tzortziou Brown V., 2016	ORB							✓	
Urquhart C., 2009	ORB							✓	
van Driel ML., 2016	ORB							✓	
van Ginneken N., 2013	ORB							✓	
van Straten A., 2009	PB	✓			✓		✓		

Study ID (Author, Year)	Type of bias assessed*	Funnel plot	Begg's	Egger's	Trim and Fill	Fail-Safe-N	Reported evidence of publication bias	Cochrane (and related) risk of bias tool	Other approach
Vasilevska M., 2014	PB	✓		✓			✓		
Vaughan KS., 2013	ORB							✓	
Vernon SW., 2010	PB	✓	✓	✓					
Virk SA., 2016	PB	✓	✓	✓	✓				
Von Meyenfeldt EM., 2012	PB			✓					
Walsh CM., 2012	ORB							✓	
Warsi A., 2004	PB	✓					✓		
Watson SJ., 2016	ORB							✓	
Weeks G., 2016	PB and ORB	✓					✓	✓	
Wei I., 2011	ORB							✓	
Weller CD., 2016	ORB							✓	
Witter S., 2012	ORB							✓	
Wootton R., 2012	PB	✓					✓		

Study ID (Author, Year)	Type of bias assessed*	Funnel plot	Begg's	Egger's	Trim and Fill	Fail-Safe-N	Reported evidence of publication bias	Cochrane (and related) risk of bias tool	Other approach
Zhai YK., 2014	PB and ORB	✓		✓	✓		✓	✓	
Zhao FF., 2017	PB and ORB	✓	✓	✓				✓	
Zhou S., 2016	ORB							✓	

*ORB: Outcome Reporting Bias and PB: Publication bias
